# Structural and functional improvement of amygdala sub-regions in postpartum depression after acupuncture

**DOI:** 10.3389/fnhum.2023.1163746

**Published:** 2023-05-17

**Authors:** Xingxian Huang, Yuanyuan Zhuo, Xinru Wang, Jinping Xu, Zhuoxin Yang, Yumei Zhou, Hanqing Lv, Xiaoming Ma, Bin Yan, Hong Zhao, Haibo Yu

**Affiliations:** ^1^The Fourth Clinical Medical College of Guangzhou University of Chinese Medicine, Shenzhen, China; ^2^Acupuncture Department, Shenzhen Traditional Chinese Medicine Hospital, Shenzhen, China; ^3^Shenzhen Key Laboratory of Modern Applied Research on Acupuncture and Moxibustion, Shenzhen, China; ^4^CAS Key Laboratory of Human-Machine Intelligence-Synergy Systems, Shenzhen Institutes of Advanced Technology, Chinese Academy of Sciences, Shenzhen, China; ^5^Luohu District of Hospital of Traditional Chinese Medicine, Shenzhen, China

**Keywords:** postpartum depression, acupuncture, functional magnetic resonance imaging, amygdala sub-regions, functional connectivity, voxel-based morphometry

## Abstract

**Objective:**

This study aimed to analyze the changes in structure and function in amygdala sub-regions in patients with postpartum depression (PPD) before and after acupuncture.

**Methods:**

A total of 52 patients with PPD (All-PPD group) were included in this trial, 22 of which completed 8 weeks of acupuncture treatment (Acu-PPD group). An age-matched control group of 24 healthy postpartum women (HPW) from the hospital and community were also included. Results from the 17-Hamilton Depression Scale (17-HAMD) and the Edinburgh Postnatal Depression Scale (EPDS) were evaluated, and resting-state functional magnetic resonance imaging (rs-fMRI) scans were performed at baseline and after the acupuncture treatment. Sub-regions of the amygdala were used as seed regions to measure gray matter volume (GMV) and analyzed for resting-state functional connectivity (RSFC) values separately. Finally, correlation analyses were performed on all patients with PPD to evaluate association values between the clinical scale scores, GMV, and RSFC values, while controlling for age and education. Pearson's correlation analyses were conducted to investigate the relevance between GMV and RSFC values of brain regions that differed before and after acupuncture treatment and clinical scale scores in Acu-PPD patients.

**Results:**

The HAMD scores for Acu-PPD were reduced after acupuncture treatment (*P* < 0.05), suggesting the positive effects of acupuncture on depression symptoms. Structurally, the All-PPD group showed significantly decreased GMV in the left lateral part of the amygdala (lAMG.L) and the right lateral part of the amygdala (lAMG.R) compared to the HPW group (*P* < 0.05). In addition, the GMV of lAMG.R was marginally increased in the Acu-PPD group after acupuncture (*P* < 0.05). Functionally, the Acu-PPD group showed a significantly enhanced RSFC between the left medial part of the amygdala (mAMG.L) and the left vermis_6, an increased RSFC between the right medial part of the amygdala (mAMG.R) and left vermis_6, and an increased RSFC between the lAMG.R and left cerebelum_crus1 (*P* < 0.05). Moreover, correlation studies revealed that the GMV in the lAMG.R was significantly related to the EPDS scores in the All-PPD group (*P* < 0.05).

**Conclusion:**

Our findings demonstrated that the structure of amygdala sub-regions is impaired in patients with PPD. Acupuncture may improve depressive symptoms in patients with PPD, and the mechanism may be attributed to changes in the amygdala sub-region structure and the functional connections of brain areas linked to the processing of negative emotions. The fMRI-based technique can provide comprehensive neuroimaging evidence to visualize the central mechanism of action of acupuncture in PPD.

## 1. Introduction

Postpartum depression (PPD) is a subtype of depression in which women experience significant depressive symptoms from 4 weeks to 12 months after giving birth, which may have an impact on both the mother's life and the parenting of her offspring (O'Hara and McCabe, 2013). Low spirits, a lack of pleasure, sleep and eating disorders, timidity and fear, anxiety and anger, a loss of self-care and baby care, and even suicidal ideation are all common symptoms of PPD (Stewart and Vigod, 2019). The global prevalence of PPD is 12–19% (Bener et al., 2012; Shorey et al., 2018). Postpartum suicide accounts for up to half of all perinatal suicides, making it the main cause of perinatal death (Chin et al., 2022). Psychotherapy and medication make up the majority of the treatment (Curry et al., 2019); however, psychotherapy works better for low to moderate postpartum depression with limited short-term impact, and there are side effects of antidepressants, such as drowsiness, dizziness, and occasionally lack of concentration. Therefore, many patients resist and interrupt their medications, thus reducing their efficacy. It is also worth noting that mothers are concerned about taking psychotropic drugs because of the need for breastfeeding (Šebela et al., 2019). Therefore, PPD is regarded as a major global issue of public health which is in desperate need of alternative therapy.

Acupuncture, as a green, convenient, safe, and effective treatment method, shows the prospect of wide application, and has been used as an alternative therapy for patients with depression (Li et al., 2019; Luo et al., 2022). It can play a role in alleviating depressive symptoms by toning down emotions and harmonizing qi and blood (Yan et al., 2021). Nevertheless, the mechanisms of action behind the beneficial effects of acupuncture for PPD are controversial, and there is still a lack of strong evidence for its effectiveness and safety (Yang et al., 2018; Hu et al., 2022).

Resting-state functional magnetic resonance imaging (rs-fMRI) is a non-invasive technique to study whole-brain resting-state functional connectivity (RSFC) and can be utilized to explore FC changes in different brain areas by assessing changes in the ratio of oxyhemoglobin and deoxyhemoglobin (Logothetis et al., 2001). It has been widely used to quantify the activating and modulating effects of acupuncture on specific brain areas (Feng et al., 2011; Cai et al., 2018). Compared with HPW, patients with PPD showed significantly increased fractional anisotropy (FA) and axial diffusivity (AD) in the right thalamic tract and FA in the cerebellar tract by DTI measurements, suggesting damage to cortico-thalamic circuits (Long et al., 2022). Patients with PPD have significantly stronger functional connectivity in the right hippocampus with the left precuneus and left superior frontal gyrus (SFG) and significantly decreased functional connectivity in the left dorsolateral prefrontal cortex (dlPFC) and right insula (Li et al., 2023). Moreover, previous studies have indicated that PPD is associated with a reduced response of the amygdala to negative stimuli and abnormalities in its structural and functional connectivities (Wonch et al., 2016; Mao et al., 2020; Kim et al., 2021). The amygdala forms part of the limbic system, which can translate somatosensory stimuli, such as those of acupuncture, into emotional states (Hui et al., 2010).

Based on the preceding research, we hypothesized that the amygdala is associated with postpartum depression; nevertheless, the mechanisms by which the amygdala sub-regions are involved in emotional processing and responses to acupuncture remain unclear. The following are our study hypotheses. First, there may be structural/functional damage in the amygdala sub-regions of patients with PPD compared to healthy postpartum women (HPW). Second, effective acupuncture treatment improves the structure/function of the amygdala sub-regions in patients with PPD. Therefore, the study's objective was to investigate the alternations in structural and FC of the amygdala sub-regions in the resting state before and after acupuncture in HPW and patients with PPD, using functional magnetic resonance imaging (fMRI), to aid in the diagnosis and clinical treatment of PPD with acupuncture.

## 2. Materials and methods

### 2.1. Study participants

From June 2019 to June 2022, all participants were enrolled via advertisements at the Shenzhen Traditional Chinese Medicine Hospital. PPD was diagnosed using the Diagnostic and Statistical Manual of Mental Disorders, Fifth Edition (DSM-V). The inclusion criteria for patients with PPD were as follows: (1) patients with PPD diagnosed by a psychiatrist; (2) patients with onset in < 12 months of delivery; (3) patients with Hamilton depression assessment scale (17-HAMD) scores ranging from 7 to 24, the Edinburgh Postnatal Depression Scale (EPDS) scores >13; and (4) patients who agreed to undergo acupuncture treatment. Patients were excluded from the study if they met any one of the following criteria: (1) patients with DSM-V-diagnosed bipolar disorder or some serious mental disorders, such as schizophrenia; (2) patients with dysnomia or inability to understand the questionnaire; (3) patients with a score of more than 2 on the “suicide” item in the 17-HAMD; (4) patients who attempted suicide within the previous year; or (5) patients with contraindications to MRI. An age-matched group of HPW was also recruited with the following inclusion criteria: patients with (1) absence of overt discomfort; (2) normal postpartum health examination (routine blood count, liver function, renal function, and electroencephalogram results); (3) 17-HAMD score < 7; and (4) no contraindications to MRI. HPW was subject to the same exclusion criteria as patients with PPD. Finally, 52 patients with PPD and 24 HPW were included in the study, of which 22 patients with PPD completed acupuncture treatment.

The Ethics Committee of the Shenzhen Traditional Chinese Medicine Hospital [Shenzhen, China; Registration No. (2018), 81] approved this study. The Helsinki Declaration was adhered to strictly in the design and execution of every therapy. Eligible participants were informed of all procedures, benefits, and potential risks that they might face in this trial, and they could quit the study at any time for any reason. Prior to participating in the trial, all individuals signed written informed consent forms. These were for participation in the study and publication.

### 2.2. Clinical measurements

All the participants completed the 17-HAMD and the EPDS assessments. The total score of the EPDS ranges from 0 to 30, with a score above 13 indicating clinically significant depression.

### 2.3. Acupuncture treatment

**Figure 1:**
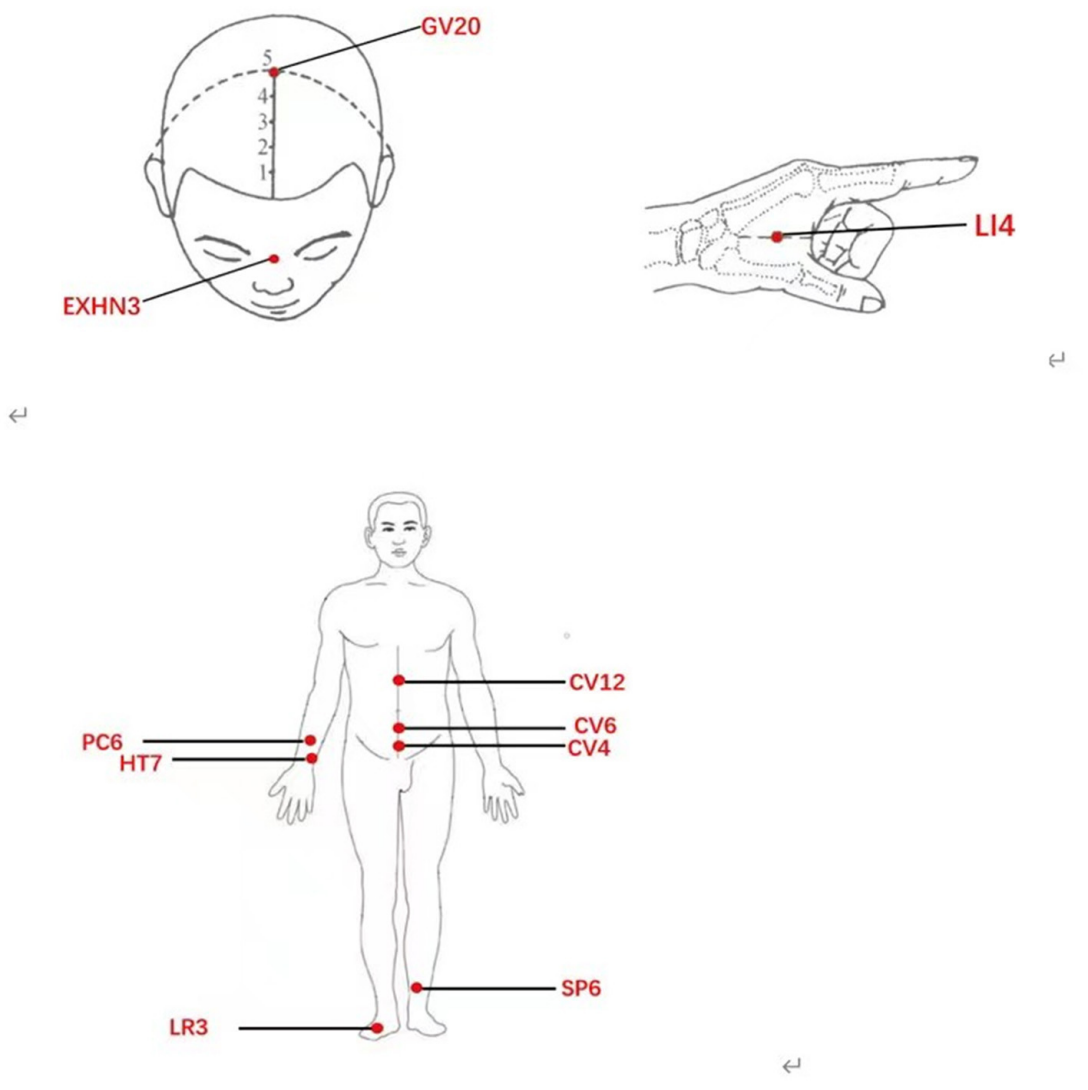
Acupoints used in this trial.

Acupuncture treatment for patients with PPD will initiate within 1 week after screening by professional acupuncturists. The acupuncturists used the “Tiao Ren Tong Du” method of acupuncture. Baihui (GV20), Yintang (EX-HN3), Zhongwan (CV12), Qihai (CV6), Guanyuan (CV4), Neiguan (PC6), Shenmen (HT7), Hegu (LI4), Sanyinjiao (SP6), and Taichong (LR3) were selected as acupoints (Figure 1). Each patient who met the inclusion criteria used the same 10 acupuncture points, positioned with reference to the World Health Organization (WHO) acupuncture point positioning method. To induce a sensation of soreness (de qi), needles will be inserted horizontally at a range of 0.5–1.2 cun and rotated and lifted for 30 s. Then, over the next 30 min, the acupoints Baihui (GV20), Yintang (EX-HN3), Zhongwan (CV12), and Qihai (CV6) will each receive electrical stimulation with a continuous wave and a low frequency of 2 Hz, while other acupoints will receive manual stimulation every 10 min by rotating or elevating the needles. Furthermore, the acupuncturists will modify the intensity according to the tolerance of the patients. The same acupuncture prescription was given for each patient. All these steps were performed by the same acupuncturist for each patient three times per week for 8 weeks, and we have three experienced acupuncturists (WL, YL, and ZY) for all patients.

### 2.4. MRI acquisition

Functional imaging data were scanned using a 3.0 Tesla MRI scanner (Siemens MAGNETOM Prisma 3.0 T) in the Shenzhen Traditional Chinese Hospital. During scanning, participants were required to relax with their eyes closed, refrain from thinking about anything specific, and avoid dozing off. Functional images were acquired using an echo-planar imaging (EPI) sequence with the following parameters: repetition time = 2000 ms, echo time = 30 ms, flip angle = 90°, the field of view = 220 × 220 mm^2^, matrix = 64 × 64, slice thickness = 3.2 mm, slice number = 37, and 240 volumes. T1 images were acquired using Magnetization-Prepared Rapid Gradient-Echo (MPRAGE) with repetition time = 2200 ms, echo time = 2.45 ms, matrix = 256 × 256, flip angle = 8°, a voxel size of 1 mm × 1 mm × 1 mm, and 178 slices.

### 2.5. MRI preprocessing

Utilizing the Data Processing and Analysis of Brain Imaging toolbox (DPABI v6.0, http://lab.rfmri.org/), the T1-weighted images of each subject were preprocessed. First, the visual quality of each image was examined; no participant was disqualified for having low imaging quality. The new-segment and DARTEL components of a one-step standard pipeline were used to process the T1 images. All of the images were converted into the typical Montreal Neurological Institute (MNI) space after being divided into three tissues: gray matter, white matter, and cerebrospinal fluid. These images were then modulated in order to maintain the regional volume information. For further morphological studies, the modulated gray matter images were smoothed with a 6-mm full-width at half maximum. The fMRI images were preprocessed using DPABI. The major procedures were as follows: (1) the first 10 functional images were eliminated; (2) subsequent images were corrected by slice timing and realignment, those with excessive head motion (>3 mm of displacement or >3° of rotation) were excluded; (3) then functional images were spatially normalized to MNI space by using EPI template; these transformation parameters were then applied to the functional images, which were resliced at a resolution of 3 × 3 × 3 mm^3^; (4) smoothing was accomplished using a Gaussian kernel with full width and half height of 8 mm; (5) Friston-24 head motion parameters, white matter, and cerebrospinal fluid signals, as well as the global mean signals were regressed; (6) temporal band-pass filtering was applied (0.01–0.1 Hz); and (7) scrubbing of bad images (two-time points before and one-time point after) that exceeded the pre-set criterion [frame displacement (FD) < 0.5 mm] for excessive motion.

### 2.6. Definition of amygdala sub-regions

**Figure 2:**
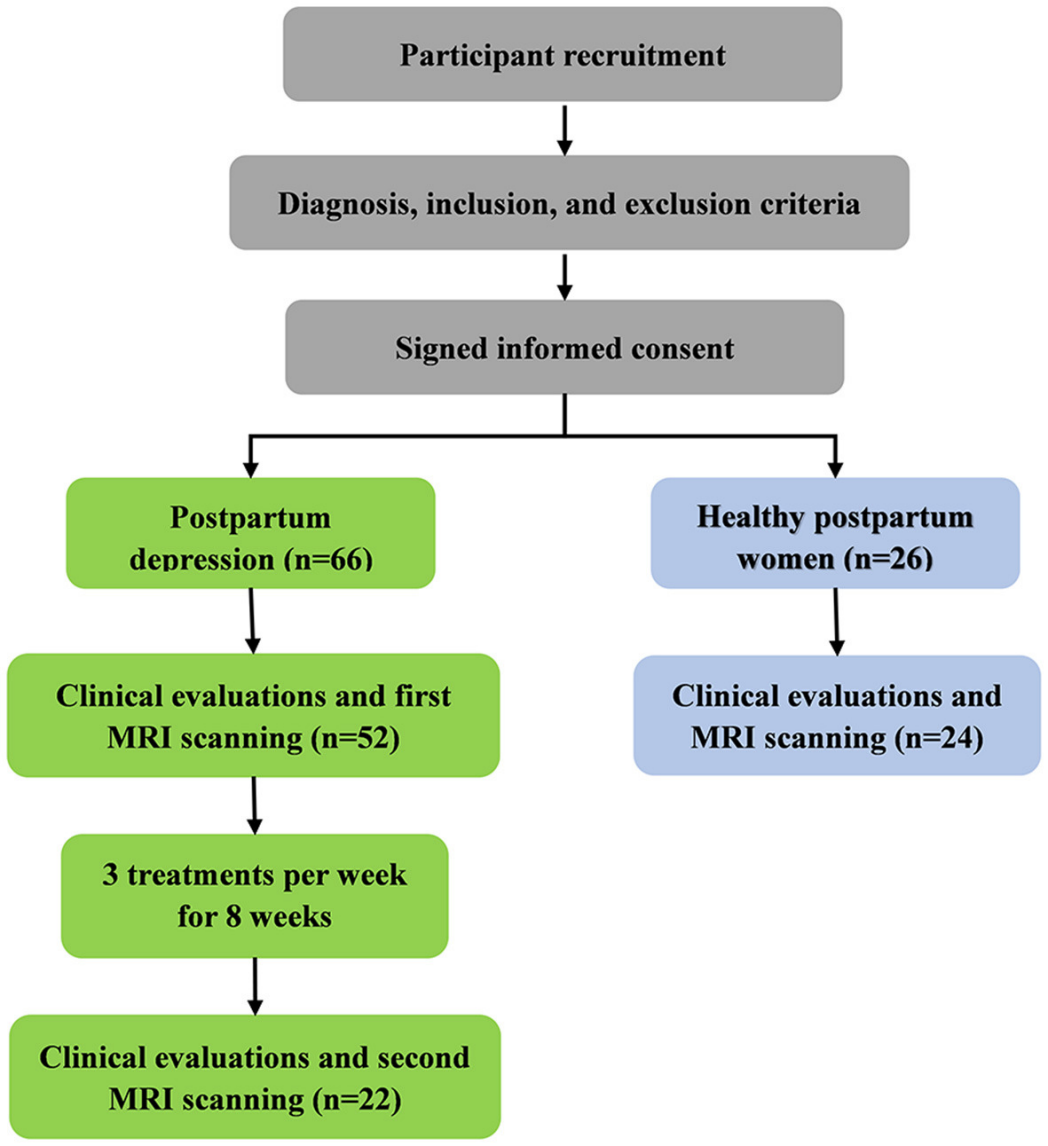
Flow chart of the study design.

The amygdala sub-regions were obtained from the Brainnetome atlas (http://atlas.brainnetome.org/) and included the left medial part of the amygdala (mAMG.L), the right medial part of the amygdala (mAMG.R), the left lateral part of the amygdala (lAMG.L), and the right lateral part of the amygdala (lAMG.R) (Figure 2). Then, these four masks were resampled to 1.5 × 1.5 × 1.5 mm^3^ for structural analyses and 3 × 3 × 3 mm^3^ for fMRI analyses.

### 2.7. Differences in GMV

The mean value of each amygdala sub-region in the modulated and smoothed gray matter maps was used to measure the GMV. Then, with age and educational background as confounding factors, two-sample *t*-tests were managed to perform between all patients with PPD and HPW, and paired *t*-tests were performed for the PPD group before and after acupuncture. *P* < 0.05 was used as the significance level.

### 2.8. The difference in RSFC

The Pearson's correlation coefficients between the mean time series of each amygdala sub-region and those of eTach voxel in the rest of the brain were applied to calculate the RSFC. Then, in order to increase normality, Fisher's z transformation was used to convert each correlation coefficient to a z value. Two-sample *t*-tests were performed between all patients with PPD and HPW, with age and education as covariates, and paired *t*-tests were performed for the PPD group before and after acupuncture. The results were corrected using Gaussian random theory, with a voxel level of *P* < 0.01 and a cluster level of *P* < 0.05. All these steps were performed using DPABI.

For regions that showed different RSFC results, we also calculated the regional mean RSFC. Then, two-sample *t*-tests were performed for all patients with PPD and HPW, with age and education as covariates, and paired *t*-tests were performed for the PPD group before and after acupuncture. The significance levels were set at *P* < 0.05.

### 2.9. Correlation analyses

Correlation analyses were performed to evaluate associations between the clinical scale scores in All-PPD groups and the GMV and RSFC values while controlling for age and education. Moreover, correlation analyses were also carried out between changed regional GMV, RSFC values, and HAMD scores in the Acu-PPD group. The statistical significance level was set at a *P*-value of < 0.05.

### 2.10. Statistical analyses

SPSS software, package version 24.0 (SPSS Inc, Chicago, IL, USA), was used to evaluate clinical data. To investigate the differences in the demographics and clinical characteristics, two-sample *t*-tests or Mann–Whitney U-tests were performed for age, education level, EPDS scores, HAMD scores, GMV, and RSFC between All-PPD and HPW, as well as between Acu-PPD patients and HPW after Shapiro–Wilk normality testing. Paired *t*-tests were used for HAMD scores for Acu-PPD patients before and after treatment. Statistical significance was set at a *p*-value of < 0.05.

## 3. Results

### 3.1. Demographic data

The flow chart of this study is shown in Figure 2.

Finally, 52 patients with PPD and 24 HPW were included, with 22 patients with PPD completing acupuncture treatment. There were no age and education differences between all the All-PPD and HPW groups or between the Acu-PPD and HPW groups. The EPDS and HAMD scores were significantly lower in the HPW, compared to those of the All-PPD and Acu-PPD groups (*P* < 0.05) (Table 1). Moreover, the HAMD scores were significantly decreased after acupuncture in the Acu-PPD group, suggesting the positive effects of acupuncture on depression symptoms.

**Table 1:** Characteristics of participants.

	**HPW**	**All PPD**	**Acupuncture PPD before**	**Acupuncture PPD after**	***P-*values^a^**	***P-*values^b^**	***P-*values^c^**	***P-*values^d^**
Numbers	24	52	22	22	-	-	-	-
Age	32.41 ± 4.26	32.73 ± 3.93	32 ± 3.92	32 ± 3.92	0.753	0.198	0.198	-
Education	16.04 ± 2.49	15.28 ± 2.29	15.72 ± 1.75	15.72 ± 1.75	0.083	0.297	0.297	-
EPDS	4.37 ± 3.24	15.51 ± 5.24	16.45± 6.09	-	< 0.001	< 0.001	< 0.001	-
HAMD	3.75 ± 1.89	14.09 ± 3.76	15.13 ± 3.57	8.09 ± 4.39	< 0.001	< 0.001	< 0.001	< 0.001

^a^P-values between All-PPD and HPW; ^b^P-values between acupuncture PPD before and HPW; ^c^P-values between acupuncture PPD after and HPW. As the education and EPDS scores were not normally distrusted, the between-group difference was performed using Mann–Whitney U-tests, whereas others used two-sample t-tests. ^d^P-values between acupuncture PPD before and after using paired t-test. Values are means ± standard deviations. HPW, healthy postpartum women; PPD, postpartum depression; EPDS, Edinburgh postnatal depression scale; and HAMD, Hamilton depression scale.

### 3.2. Gray matter volume

**Figure 3:**
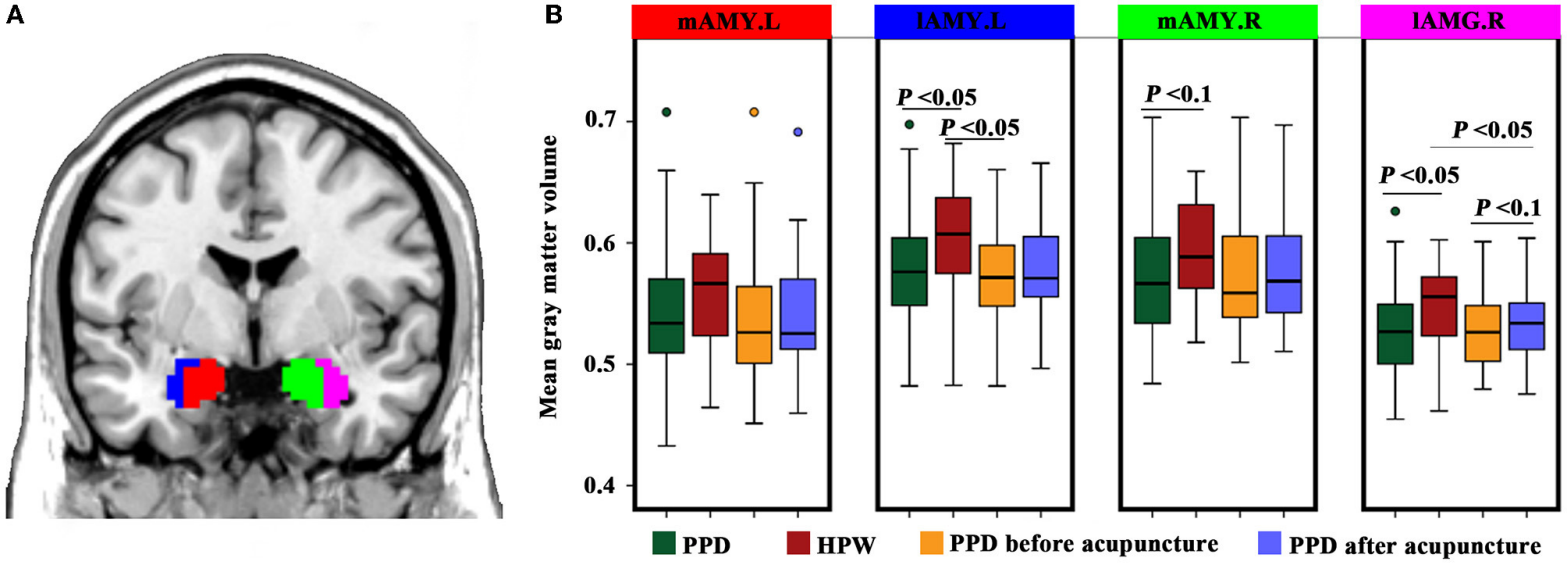
Structural changes in the amygdala sub-regions. **(A)** Definitions and locations of amygdala sub-regions. The amygdala sub-regions were obtained from the Brainnetome atlas (http://atlas.brainnetome.org/), and include the left medial part of the amygdala (mAMG.L, red), right medial part of the amygdala (mAMG.R, green), left lateral part of the amygdala (lAMG.L, blue), and right lateral part of the amygdala (lAMG.R, violet). **(B)** Gray matter volume changes for amygdala sub-regions in patients with PPD compared to healthy postpartum controls, using two-sample *t-*tests, as well as in the patients with PPD after acupuncture compared to those before acupuncture using paired *t*-tests. A *P*-value of < 0.05 was considered significant, and a *P*-value of <0.1 was considered marginally significant.

Compared to the HPW, the All-PPD group showed significantly decreased GMV in the lAMG.L and lAMG.R (*P* < 0.05). Moreover, the GMV of the lAMG.R was marginally increased in the Acu-PPD group (*P* < 0.05) (Figure 3).

### 3.3. Results of the RSFC

**Figure 4:**
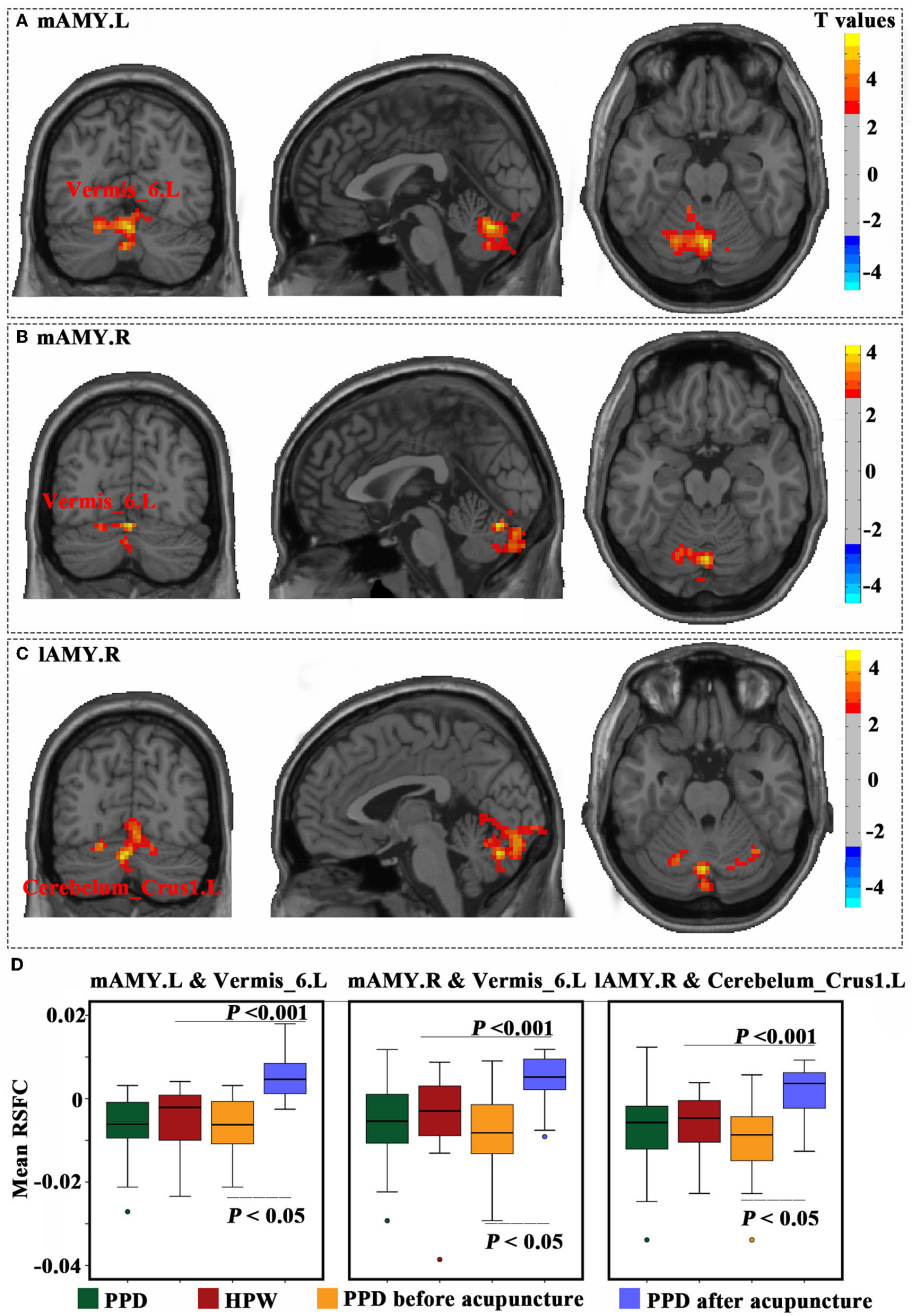
Functional connectivity changes in the amygdala sub-regions. The results of the left medial part of the amygdala **(A)**, right medial part of the amygdala **(B)**, and right lateral part of the amygdala **(C)** were obtained using paired *t*-tests of patients with PPD before and after acupuncture. These results were corrected using a Gaussian random field with a voxel level of *P*< 0.01, and a cluster level of *P* < 0.05. **(D)** Mean resting-state functional connectivity was calculated for all four groups, and compared using two-sample *t*-tests between patients with PPD and healthy postpartum women, as well as paired *t*-tests between patients with PPD before and after acupuncture. A *P*-value of < 0.05 was considered significant.

The Acu-PPD group showed a significantly increased RSFC between the mAMG.L and left vermis_6, an increased RSFC between the mAMG.R and left vermis_6, and an increased RSFC between the lAMG.R and left cerebelum_crus1 (*P* < 0.05) (Figure 4). However, no significant differences were identified in RSFC results between the All-PPD and HPW groups for all four amygdala sub-regions.

### 3.4. Results of correlation analysis

**Figure 5:**
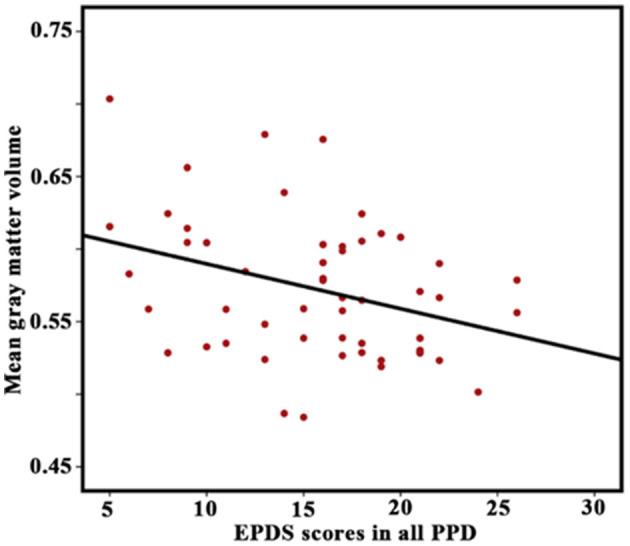
Correlation between Edinburgh Postnatal Depression Scale (EPDS) scores in all patients with PPD and gray matter volume in the right lateral part of the amygdala. A *P*-value of < 0.05 was considered significant.

Correlation analyses showed that the GMV in the lAMG.R was significantly correlated with EPDS scores in the all-PPD group (*P* < 0.05) (Figure 5). However, no significant result was identified between HAMD scores and any altered structural and functional indexes.

## 4. Discussion

### 4.1. Acupuncture is becoming the main stream in medicine

Acupuncture has been accepted by a growing number of Western nations due to studies showing that it is safe and helpful in treating postpartum depression. Moreover, acupuncture can enhance the effects of drug treatment of PPD, relieving depressive symptoms (Wong et al., 2021; Zhang et al., 2021; Yang et al., 2022). A recent meta-analysis included six systematic reviews/meta-analyses of acupuncture for PPD to assess its effectiveness. Three of them reported that acupuncture was more effective than controls, and three found no difference, making it difficult to draw consistent conclusions (Hu et al., 2022). In summary, the current study confirms that acupuncture has some efficacy in the treatment of PPD, but the mechanism is unclear, and the evidence is insufficient. As far as we know, this is the first fMRI study that demonstrates that acupuncture improves structural and functional connectivity in the amygdala sub-regions of the brain in patients with PPD. Our results confirm our hypotheses that, first, the structure of the amygdala sub-region is impaired in patients with PPD, and second, that acupuncture can remodel the structure and function of the amygdala sub-region in these patients. In conclusion, the change in the volume and functional connections of the amygdala sub-regions may be a potential therapeutic target for acupuncture in the treatment of postpartum depression.

### 4.2. Acupuncture modulates the structure of the amygdala sub-regions

In our study, the GMV of the amygdala sub-regions of lAMG.L and lAMG.R was smaller in patients with PPD compared to HPW, while lAMG.R's GMV was somewhat increased in patients with PPD after acupuncture. Correlation analysis of lAMG.R GMV with the EPDS scores in patients with PPD revealed a negative correlation, indicating that the material basis for the altered brain function in patients with PPD may be closely related to abnormal amygdala gray matter density from the perspective of brain structure. Since the amygdala is one of the neuroimaging biomarkers linked to the response to depression (Fonseka et al., 2018), our findings may help to improve the knowledge of the distinctive characteristics and functional connections of the amygdala sub-regions.

Numerous studies have employed voxel-based morphometry (VBM) to measure the structural alterations in the brain linked to depression (Ashburner and Friston, 2000). The limbic system, consisting of the hippocampus and amygdala, is the material basis for emotion production (Rolls, 2019). The amygdala is an essential structure for the generation, recognition, and regulation of emotions (especially fear), which is anatomically heterogeneous, consisting of a complex of 13 nuclei of variable size. Additionally, the sub-regions of the amygdala are thought to have different functions and transmit signals from multiple brain regions (Sah et al., 2003). The amygdala is activated when processing baby-related stimuli, which is associated with nurturing behavior, and the increase in the GMV in the amygdala may contribute to mother–infant interactions (Kim et al., 2010).

The basolateral amygdala (BLA) and the central medial amygdala (CMA) are two major components of the amygdala that are thought to play different roles in emotional functions and internal states as core nodes of emotion-related brain circuits (Zeng et al., 2021). The BLA is the amygdala's main input structure, responsible for receiving afferent signals from sensory cortical areas, including visual and auditory inputs, and plays a big part in threat learning and associated plasticity (Sears et al., 2014; Adhikari et al., 2015). The amygdala's output is primarily derived from the CMA and is associated with behavior, the autonomic nervous system, and the endocrine system (Sah et al., 2003). Additionally, previous research has suggested a lateralized difference in the right amygdala, implying that depression is more closely associated with the right amygdala than the left, which is consistent with our findings (Kim et al., 2021; O'Brien et al., 2021). In fact, a clinical study found that stimulating the right amygdala induces more negative emotions, such as fear and sadness, and rapid processing of emotions, while stimulating the left amygdala induces more positive emotions and may be associated with long-term sustained stimulation (Lanteaume et al., 2007). Some studies hypothesized that the right amygdala plays a role in retrieving pertinent information and that it is more readily triggered by visual stimuli than the left side (Markowitsch and Staniloiu, 2011). The total volume of the amygdala and its sub-regions (including the right lateral nucleus, the anterior region of the right amygdala, and the entire right amygdala) were significantly smaller in depressed patients than in healthy controls (Bruder et al., 2017; Caetano et al., 2021), and the lateral nucleus and anterior region of the right amygdala were correspondingly smaller when the depression was more severe (Brown et al., 2020; Okamoto et al., 2022).

Electroconvulsive therapy (ECT), a highly effective treatment for refractory depression, is thought to be excellent for neuroplasticity (Cai et al., 2022). Several studies have found that electroconvulsive therapy causes a significant increase in GMV in the right amygdala (Gryglewski et al., 2019, 2021; Camilleri et al., 2020). Our previous study confirmed that ECT can selectively increase the volume of the hippocampal-amygdala transition region bilaterally in patients with depression (Xu et al., 2022). In the current study, we found that the change in GMV was directly correlated with acupuncture treatment, which has the same effects as ECT while being green, convenient, safe, and highly effective. As a result, we can propose that the change in GMV in the right lateral nucleus of the amygdala is reversible, that acupuncture can increase the GMV in the right lateral nucleus of the amygdala and relieve clinical symptoms, and that the plastic change in GMV in the right lateral nucleus of the amygdala may be related to the mechanism of acupunctures efficacy for postpartum depression. Few studies have been performed to explore the relationship between amygdala sub-region volume changes and acupuncture treatment. Previous research has shown that acupuncture can cause significant structural reorganizations of brain regions. For instance, Wu et.al discovered an increase in gray matter structure in motion-related areas in patients with ischemic stroke treated with acupuncture for 4 weeks, using VBM analysis (Wu et al., 2018). Yang et al. (2020) collected data on 41 patients with migraine and found that 19 of them responded to acupuncture. They also found that the increase in GMV in the relevant brain region was associated with a decrease in migraine severity (Yang et al., 2020). In conclusion, GMV in the amygdala sub-region may become a new biomarker of testing acupuncture efficacy.

### 4.3. Acupuncture regulates functional connectivity in the amygdala sub-region

Depression is increasingly understood as a disorder of connectivity between brain networks (Drevets et al., 2008). Previous research showed dysfunction in brain regions associated with cognitive and emotional control in patients with PPD, including the cingulate gyrus, temporal lobe, frontal lobe, amygdala, hippocampus, parietal lobe, and occipital lobe (Guo et al., 2014; Mao et al., 2020). The amygdala is the most well-studied and extensive of these, although previous studies assessing amygdala activity in depressed patients have frequently reached different and even contradictory conclusions. Because of the heterogeneity of amygdala structure and function, PPD studies have increasingly concentrated on the RSFC of the amygdala sub-regions.

The RSFC of the amygdala sub-regions did not differ between HPW and patients with PPD based on the available imaging data, which may be due to false-negative results in a small sample size. Thus, further research is required to confirm our findings and to obtain a deeper understanding of the neural network connections underlying the amygdala in PPD. Studies have shown impaired functional connections in brain regions involved in maternal care networks in PPD compared to HPW (Zhang et al., 2020). Laurent and Ablow (2012) exposed 11 patients with PPD and 11 HPW to infant cries and found that those with PPD exhibited retarded activation of the amygdala with impaired motivational responses to infants (Laurent and Ablow, 2012). Prior research has suggested that in patients with PPD, right amygdala-posterior cingulate cortex (PCC) connections are significantly disrupted when compared to healthy mothers. PCC is seen as a concern for responsibility, and disruption of the PCC amygdala may be related to the mother's failure to adapt to her responsibility (Chase et al., 2014).

Nevertheless, to our surprise, we found that functional brain connections between the mAMG.L and left vermis 6, mAMG.R and left vermis 6, as well as lAMG.R and left cerebellum crus1 were strengthened after acupuncture. From this, we hypothesize that PPD treated with acupuncture resulted in the activation of neural activity and enhanced amygdala-cerebellar connectivity, which led to an increased ability of the mother to regulate negative emotions and an enhanced motivational response to the infant. Therefore, the repair of the amygdala-cerebellar circuit may be potential evidence for the effectiveness of acupuncture.

In the postpartum period, the amygdala is strongly activated by signals from the infant, and it plays a key role in activating motivation, reward systems, and emotion regulation (Dufford et al., 2019). Thus, the regions connected to the amygdala make a major contribution to supporting maternal behavior and rearing offspring. Previously, the cerebellum was thought to be important in motor control and balance regulation. However, there is mounting evidence that the cerebellum is also involved in the regulation of emotional processing, particularly negative emotional memory (Guo et al., 2013). Cerebellar electrical stimulation can elicit responses to brain regions involved in emotional regulation and cognitive processing, namely the amygdala, hippocampus, and anterior cingulate cortex, which were considered the neural basis for cerebellar-brain functional connectivity (Heath et al., 1978). Previous fMRI research has also revealed that negative emotions are connected to the right hemisphere of the brain, while positive emotions are associated with the left (Hakamata et al., 2017). When confronting negative emotions, activation of the cerebellum may be associated with activation-related action preparation (Schraa-Tam et al., 2012).

Habas et.al proposed that the cerebellar–amygdala network plays a role in sensorimotor, emotion regulation, and motivational integration by performing resting-state brain function image analysis on 15 healthy volunteers (Habas, 2018). Recent experimental animal studies using neuronal anatomical tracing and photophysiology have elaborated on the existence of a bilateral circuit between the cerebellum and the amygdala, with thalamic axons acting as key nodes receiving input from the cerebellum and thus afferent to BLA neurons (Jung et al., 2022). When faced with fear and threat, the amygdala and cerebellum are activated simultaneously, while depressed patients show impaired cerebellar–amygdala connections, which may be related to impaired fear processing (Roy et al., 2009). Tang et al. (2019) performed rs-fMRI of the amygdala sub-regions in depressed patients and healthy controls and found reduced connectivity between the centromedial (CM) nuclei group and the superficial (SF) nuclei group of the amygdala and the cerebellum in depressed patients, but no structural abnormalities of the amygdala were seen in either group, so it was hypothesized that the abnormal connectivity function was not structurally related (Tang et al., 2019).

Numerous studies showed that the amygdala might be involved in the effects of acupuncture treatment, but few were focused on the interaction between acupuncture and specific amygdala sub-regions (Qin et al., 2008; Wang et al., 2016; Duan et al., 2020). The modulatory effects of acupuncture treatment on each amygdala sub-region were shown to differ in our study, which is consistent with the findings of Wang et al.'s (2016) study, that is, the degree of response to acupuncture differed between the two sides of the amygdala in patients with depression treated with acupuncture for 8 weeks, and the changes of RSFC in the left amygdala were negatively correlated with the severity of depressive symptoms in patients treated with acupuncture for 8 weeks (Wang et al., 2016). The study found that 12 weeks of antidepressant treatment enhanced functional brain connectivity in the right CM, SF, and laterobasal (LB) nuclei group in depressed patients, resulting in symptom relief. In other words, effective connectivity in the right amygdala sub-regions predicted symptom improvement after depressive medication (Xiao et al., 2021). We speculate that the differences above are related to the different treatments and the heterogeneity of the population.

We can speculate that this modulatory effect may represent, at least in part, a potential mechanism for acupuncture's effect on postpartum depression. This is presumably due to the repair of microstructural damage in the relevant brain regions, which enhances the amygdala's function in regulating mood when patients with PPD pick up infant signals, promotes cerebellar–amygdala functional connectivity, increases sensitivity to infants, encourages mother–infant interactions, and thus improves depressive symptoms. Few studies have been conducted on cerebellar–amygdala sub-regions in patients with PPD. Future research should concentrate on the RSFC in the amygdala sub-region-cerebellar region, which may assist us in comprehending how acupuncture for PPD works.

### 4.4. Limitations

The current study still has several limitations in it. First of all, given that this is a preliminary study, the findings need to be validated further. Based on the most recent imaging data, we did not discover any differences in RSFC in the amygdala sub-region between HPW and individuals with PPD. It could be the relatively small sample size with high heterogeneity, which led to false-negative results. In light of this, it is recommended that future research review the current findings using a larger sample size. Second, there was no long-term follow-up in the current study to track the long-term effects and efficacy of follow-up acupuncture on the brain. Despite these limitations, our current study indicates that acupuncture has a modulating effect on structural and functional connectivity changes in PPD patients, providing definitive and objective evidence.

## 5. Conclusion

Our study concludes that acupuncture may help alleviate symptoms in patients with PPD and may be linked to increased brain GMV in the amygdala sub-regions and improved functional connections between the bilateral amygdala and cerebellum. Future research to explore the underlying mechanisms of action of acupuncture in PPD will need larger clinical studies with longer intervention times. These findings provide references and fresh concepts for potential clinical applications in the future, as well as an in-depth explanation of the mechanism of action of acupuncture in the treatment of PPD.

## Data availability statement

The raw data supporting the conclusions of this article will be made available by the authors, without undue reservation.

## Ethics statement

The studies involving human participants were reviewed and approved by the Ethics Committee of Shenzhen Traditional Chinese Medicine Hospital [Shenzhen, China; Registration No. (2018), 81]. The patients/participants provided their written informed consent to participate in this study.

## Author contributions

XH and ZY acquired the data. BY and XM performed the acupuncture treatment. YZhu and XW prepared this manuscript. XH and JX revised the manuscript. YZho performed the scale evaluation. HL performed the MRI scanning. HZ and HY have seen, and can confirm, the authenticity of the raw data. All the authors have read and approved the final manuscript.
